# New method for obtaining position and time structure of source in HDR remote afterloading brachytherapy unit utilizing light emission from scintillator

**DOI:** 10.1120/jacmp.v10i3.2983

**Published:** 2009-07-15

**Authors:** Haruna Kojima, Takashi Hanada, Shoichi Katsuta, Atsunori Yorozu, Koichi Maruyama

**Affiliations:** ^1^ Graduate School of Kitasato University Sagamihara Kanagawa; ^2^ National Hospital Organization Tokyo Medical Center Meguro Tokyo Japan

**Keywords:** high‐dose rate brachytherapy, I192r source information, source position, source time structure

## Abstract

When using a HDR remote afterloading brachytherapy unit, results of treatment can be greatly influenced by both source position and treatment time. The purpose of this study is to obtain information on the source of the HDR remote afterloading unit, such as its position and time structure, with the use of a simple system consisting of a plastic scintillator block and a charge‐coupled device (CCD) camera. The CCD camera was used for recording images of scintillation luminescence at a fixed rate of 30 frames per second in real time. The source position and time structure were obtained by analyzing the recorded images. For a preset source‐step‐interval of 5 mm, the measured value of the source position was 5.0±1.0mm, with a pixel resolution of 0.07 mm in the recorded images. For a preset transit time of 30 s, the measured value was 30.0±0.6 s, when the time resolution of the CCD camera was 1/30 s. This system enabled us to obtain the source dwell time and movement time. Therefore, parameters such as I192r source position, transit time, dwell time, and movement time at each dwell position can be determined quantitatively using this plastic scintillator‐CCD camera system.

PACS number: 87.53.Jw

## I. INTRODUCTION

Brachytherapy, a type of modern radiotherapy for cancer treatment, usually uses a high‐dose rate (HDR) I192r source. Unlike external beam sources, this source allows the delivery of a higher radiation dose to the target tissue and a lower radiation dose to the surrounding normal tissue. Therefore, accurate control of source position and time using a HDR remote afterloading unit is critical to ensure the effectiveness of the treatment. Verification of the accuracy of the unit is essential for achieving the best quality assurance (QA). The estimation of source position and time of the HDR remote afterloading unit is included in the QA test (as reported in AAPM Radiation Therapy Committee Task Group 40 (TG40)).^(1)^ The tolerance at the source position is 1 mm, and the test is conducted every week. The tolerance at the timer function is 1%, and the frequency for source exchange is selected arbitrarily or set to 3 months. Many studies have been conducted to obtain information on the source for QA checks of remote afterloading units. These studies involved the use of a film,^(2)^ a well‐type ionization chamber,^(^ ^3^ ^–^ ^4^ ^)^ a radiochromic film,^(^ ^5^ ^–^ ^6^ ^)^ and a fluorescent screen.^(7)^ Li et al.^(8)^ designed a QA test tool consisting of two concentric disks.

The purpose of this study is to obtain information on the source of the HDR remote afterloading unit, such as its source position and time structure, by using a plastic scintillator block and a charge‐coupled device (CCD) camera system. The use of the plastic scintillator in brachytherapy is a relatively new development, and dosimetric measurements have been reported in some cases.^(^ ^9^ ^–^ ^10^ ^)^ With the help of the CCD camera, it is possible to monitor the HDR remote afterloading unit in real time, and simultaneously obtain the source position and time structure in digital form. The plastic scintillator and CCD camera system has been developed for use in heavy ion beam and proton beam range measurement.^(^ ^11^ ^–^ ^12^ ^)^ We applied a method previously used for a particle beam to quantitatively acquire HDR I192r source information.

## II. MATERIALS AND METHODS

**Figure 1:**
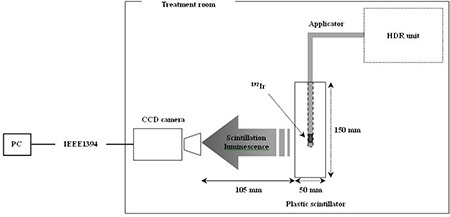
Schematic diagram of measurement system. The measurement tool consists of a block of a plastic scintillator and a CCD camera. The HDR unit delivers the I192r source through the applicator. The CCD camera records the images of scintillation luminescence and transfers the data to a PC connected to it by an IEEE 1394 cable.

Our measurement method, schematically shown in Fig. 1, is based on the method used by Fukushima et al.^(12)^ for proton beam range measurements and involves the use of a plastic scintillator block and a CCD camera. We used a block of organic plastic scintillator (Bircon, BC‐408) with dimensions of $50\times 50\times 150\;{\text{mm}}^{3}$, density of 1.032 g cm^−2^, refractive index of 1.58, rise time of 0.9 ns, and decay time of 2.1 ns. A hole (diameter of 3.2 mm) was drilled at the center of the scintillator block, for insertion of a 3 mm diameter applicator. In order to measure the source delivery information with high precision, we used a high‐definition digital video camera (SONY, HDR‐HC1) as the CCD camera. The camera was used for recording images of scintillation luminescence at a rate of 30 frames per second (fps); the recorded images were 8‐bit grayscale images. The pixel resolution of the images was 1920×1080pixels.

The HDR remote afterloading unit used in this study was a Buchler facts unit consisting of an HDR I192r stepping source. The source position in the applicator was controlled remotely, according to a preset plane moving over 5 cm with a source‐step‐interval of 5 mm and preset transit time of 30 s.

In order to analyze many images that have a resolution of 1920×1080pixels and to measure the source information at a high speed, we used automatic analysis software developed by Fukushima et al.^(12)^ The software program adds the pixel values in the region of interest (ROI) in every line of the depth direction of the plastic scintillator, to create the I192r scintillation brightness distribution. Using this software, we can measure the source position and time information from the image of scintillation luminescence.

### A. Source position and movement distance

**Figure 2:**
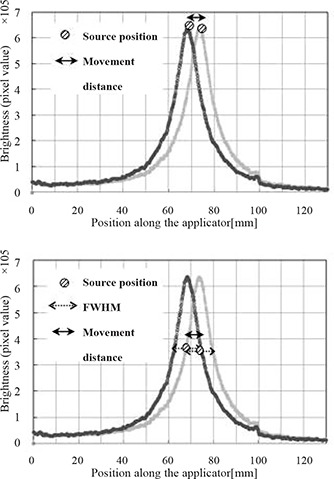
Two types of methods for source position determination. Brightness distributions at two positions are depicted: (a) one method defines the maximum brightness point as the source position; and (b) another method defines the brightness midpoint of the FWHM as the source position.

We measured the source position and movement distance from the I192r scintillation brightness distribution obtained from the scintillation luminescence images. The source position was determined with two methods that depicted the brightness distributions at two positions. One method defined the maximum brightness point at the source position, while the other method defined the brightness midpoint of the full width at half maximum (FWHM) as the source position. These two points were measured from the source position in each movement interval. Figure 2 schematically shows the two methods for the determination of the source position.

### B. Source time structure

After converting the captured scintillation luminescence images from the moving images into the frames of still images, we analyzed the sequence of frames in order to obtain the time structure of the moving source. We could obtain the I192r scintillation brightness distribution of each frame during source movement. We analyzed the distributions in order to determine the source movement time and dwell time.

We counted the number of frames required for capturing each source movement $\left(n_{M}\right)$ and calculated the source movement time $\left(t_{M}\right)$ by using the following equation, when the frame rate was fixed at 30 Hz:

(1) $$t_{M}=n_{M}\cdot 1/30$$

We summed all the movement times in order to obtain the total movement time denoted as $T_{M}$.

Similarly, we counted the number of frames required to capture the source dwell $\left(n_{D}\right)$ and calculated the source dwell time $\left(t_{D}\right)$ using the following equation:

(2) $$t_{D}=n_{D}\cdot 1/30$$

We summed all the dwell times in order to obtain the total dwell time, denoted as $T_{D}$.

Finally, we determined two types of source transit time as follows:

(3) $$T_{T}^{+}=T_{D}+T_{M}$$

(4) $$T_{T}^{-}=T_{D}$$

where $T_{T}^{+}$ is the sum of the total dwell and the total movement time and $T_{T}^{-}$ is the total dwell time.

## III. RESULTS & DISCUSSION

**Figure 3:**
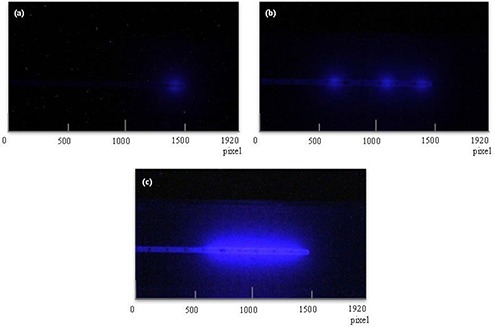
Images of scintillation luminescence. (a) Image of 1 frame when the source stops at the tip of the applicator; (b)image of 3 frames when the source movement distance was set to 5cm(2cm+3cm) in 3 steps; (c) image of 11 frames when the source movement distance was set to 5 cm in 11 steps at 5 mm intervals, where 11 frames form an image of a single bunch of scintillation luminescence.

With a plastic scintillator and a CCD camera, we were able to successfully monitor the HDR remote afterloading unit in real time and to record its images of scintillation luminescence (Fig. 3). The area of energy deposits due to interactions between photons has a high pixel value. Scintillation luminescence increased with the source in the applicator. We defined the pixel size from the recorded image. By a highly precise analysis, the pixel size was estimated as 0.069 mm/pixel.

### A. Source position and movement distance

**Figure 4:**
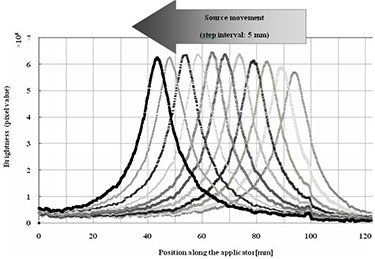
I192r scintillation brightness distributions. We obtained brightness distributions corresponding to eleven source dwell points.

**Figure 5:**
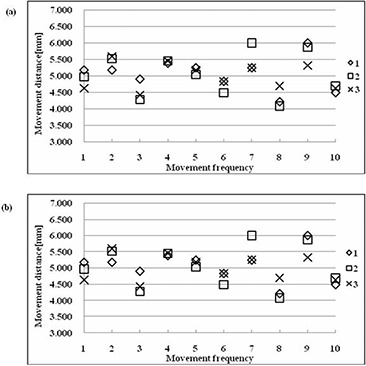
Source movement distances. Results of three sets of measurements are shown in Table 1 for the source position determined by: (a) maximum brightness point, and (b) brightness midpoint of the FWHM. The variation of all source movement distances from the preset values is less than ±1mm.

Figure 4 shows the I192r scintillation brightness distributions that were acquired from the scintillation luminescence images. Table 1 and Fig. 5 show the results of analysis of the source movement distance, which was measured from the I192r scintillation brightness distribution. For a preset source‐step‐interval of 5 mm, the measured value was 5.0±1.0mm, with a pixel resolution of 0.07 mm in the recorded images. Because the preset source‐step‐interval was 5 mm, this value satisfied the tolerance of QA for the source position of HDR remote afterloading units in TG40. Moreover, this system was highly precise. The decision precision was such that it allowed us to estimate source position and source movement distance, because this was equal to the pixel resolution (0.069 mm) of the scintillation luminescence image. The source position, defined as the midpoint of the FWHM, gave better precision than that defined as the point of maximum brightness, which gave a precision less than ±1mm.

**Table 1:** Source movement distance at each source dwell position.

*Movement Frequency*	*Source movement distance (mm*)					
	*Set 1*		*Set 2*		*Set 3*	
	*MAX*	*FWHM*	*MAX*	*FWHM*	*MAX*	*FWHM*
1	5.175	4.623	4.968	4.796	4.623	4.830
2	5.175	5.347	5.520	5.279	5.589	5.348
3	4.900	4.830	4.278	4.692	4.416	4.727
4	5.381	5.106	5.450	5.313	5.450	5.313
5	5.244	5.175	5.037	5.037	5.175	4.934
6	4.830	4.899	4.485	4.968	4.830	5.071
7	5.244	4.968	6.003	4.968	5.244	4.830
8	4.209	4.968	4.071	5.106	4.692	5.175
9	6.002	5.244	5.865	5.106	5.313	4.037
10	4.485	4.865	4.691	5.037	4.623	5.003
Average	5.07±0.50	5.00±0.22	5.04±0.66	5.03±0.19	5.00±0.41	4.93±0.37

The preset source step interval is 5 mm. Results of three sets of measurements are shown.

### B. Source time structure

**Figure 6:**
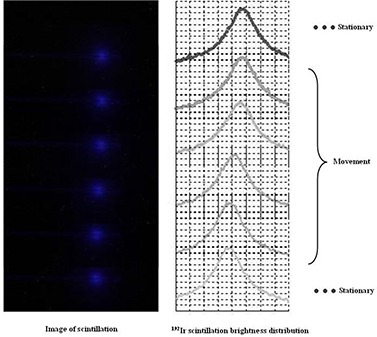
Process of source movement. The scintillation luminescence image and I192r scintillation brightness distribution changed as the source moved.

**Figure 7:**
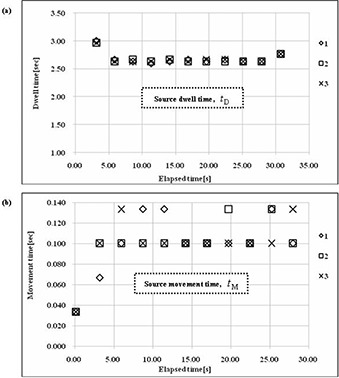
Source dwell time and source movement time obtained from the I192r scintillation brightness distributions in three sets of measurements: (a) source dwell time at each source dwell point; (b) source movement time in each source movement step.

The source time structure could be determined at a frame rate of 1/30 s using the CCD camera. Figure 6 shows the analytical results of the source movement time. The scintillation brightness distribution changed as the source moved. We could measure the source dwell time, $t_{D}$, of each dwell point and the results are shown in Fig. 7. For a preset transit time of 30 s, the measured value of the total dwell time, $T_{T}^{-}=T_{D}$, was 29.53 s; the transit time including the source movement time, $T_{T}^{+}$, was 30.60 s. The source time information is shown in Fig. 7 and listed in Table 2.

**Table 2:** Source time information.

	*1*	*2*	*3*
Source Transit Time			
Transit time $T_{T}^{+}(s)$	30.60	30.60	30.60
Transit time $T_{T}^{-}(s)$	29.53	29.50	29.53
Source Movement Time			
Each movement time $t_{M}\;(s)$	0.10~0.13	0.10~0.13	0.10~0.13
Total movement time $T_{M}\;(s)$	1.07	1.10	1.07
Source Dwell Time			
Each dwell time $t_{D}\;(s)$	2.60~3.00	2.63~2.97	2.63~2.97
Total dwell time $T_{D}\;(s)$	29.53	29.50	29.53

Preset time: 30.0 (s)

Transit time $T_{T}^{+}$ is the sum of the total dwell time and the total movement time. Transit time $T_{T}^{-}$ is the total dwell time.

Conventional measurement methods, such as those that involve the use of a stopwatch or an ion chamber, can measure only the source transit time, which is the sum of the source movement time and the dwell time. However, using our method, quantitative analysis of short‐term time‐scale variations, such as movement time and dwell time, was possible. Therefore, this method would be very useful for evaluating the time‐scale model.

The scintillation luminescence from the I192r source was recorded as digital data using a plastic scintillator and a CCD camera. We could obtain highly precise quantitative source information from the recorded images. By this method, it was possible to monitor the HDR remote afterloading unit in real time and to simultaneously obtain the source position and time structure. Moreover, this method could be used to check the drive part that sends the source out.

## IV. CONCLUSIONS

We developed a method to obtain HDR I192r source information using a plastic scintillator block and a CCD camera. By this method, we could quantitatively and simultaneously measure the source position and time structure from the recorded I192r scintillation brightness distributions. For a preset source‐step‐interval of 5 mm, the measured value was 5.0±1.0mm with a pixel resolution of 0.07 mm in the recorded images. For a preset transit time of 30 s, the measured value was 30.0±0.6 s, when the time resolution of the CCD camera was 1/30 s. Moreover, with this method, we could separate the source dwell time from the movement time.

In conclusion, precise digital information such as I192r source positions, transit time, dwell time, and movement time at each dwell position obtained by this method are useful in maintaining the level of QA of radiotherapy using the HDR remote afterloading unit.

## ACKNOWLEDGEMENTS

We would like to thank Mr. Minoru Ishigami from the Graduate School of Medical Science, Kitasato University, and staff members from the Department of Radiology, the National Hospital Organization, Tokyo Medical Center, for their help.
